# Misdiagnosis of Extensive Maxillofacial Infection and Its Relationship with Periodontal Problems and Hyperglycemia

**DOI:** 10.1155/2016/5960546

**Published:** 2016-01-14

**Authors:** Cristian Statkievicz, Leonardo P. Faverani, Pedro Henrique Silva Gomes-Ferreira, Gabriel Ramalho-Ferreira, Idelmo Rangel Garcia-Junior

**Affiliations:** Department of Surgery and Integrated Clinic, Aracatuba Dental School, Universidade Estadual Paulista (UNESP), 16015-050 Araçatuba, SP, Brazil

## Abstract

*Background*. Complex dental infections can reach distant areas of the alveolar process, invading the secondary fascial spaces.* Objectives*. This case report aims to show a misdiagnosis of odontogenic infection and a great need for dentist in the hospital environment.* Case Report*. A male patient presented facial asymmetry and trismus, while the facial CT examination showed a hyperdense mass involving the left masseteric, pterygomandibular, and superficial temporal regions. The patient was then referred to oral oncology center by emergency physician with cancer suspicion. After 15 days, the patient returned to the same emergency room and was attended by the surgical and maxillofacial trauma team, presenting tachycardia, tachypnea, dysphagia, and trismus. During anamnesis, the patient reported being an uncontrolled diabetic. In intraoral exam, a poor oral condition and generalized periodontitis were observed.* Results*. Correct diagnosis of odontogenic infection was established and adequately treated.* Conclusions*. Symptomatology bland may mask the severity of an infection; every increase in volume associated with trismus, poor oral hygiene with or without hyperglycemia should be heavily investigated for the presence of an infectious process. It emphasizes the importance of a dentist working with the physician in emergency room.

## 1. Introduction

Maxillofacial infections are usual in patients of all ages, representing a substantial risk to life when reaching deep facial spaces [1]. The signs and symptoms are evident due to proximity between nerves, muscles, and ligaments, which quickly impairs the function of affected region, leading to pain and trismus [2]. Although well-defined, facial anatomy presents peculiar ways for infection spreading, which, when invaded, communicate with each other being able to store large amounts of pus, what may cause upper airways compression and/or face asymmetry, requiring a fast and accurate treatment [3].

In general, the computed tomography (CT) assists in the identification of infection limits, which is vital for a precise diagnosis [1] and provides the surgeon conditions for appropriate surgical drainage, along with cause removal. These two steps are the basis for therapy, supported by the administration of specific antibiotics [4, 5].

Infectious processes within the oral cavity are generally associated with an inadequate oral hygiene, periodontal disease, and caries [6], being exponentially aggravated in immunosuppressed and especially diabetic patients [7]. Therefore, the dentist or doctor must keep odontogenic infection under suspicion when a painful edema is observed in face or neck [2]. Thus, a specialized consultation with a dentist or an oral and maxillofacial surgeon can avoid serious complications and save lives [2].

Although it is hard to determine whether an infection will get worse at the moment the patient is admitted for treatment [6], the triad anamnesis, physical examination, and image tests should be performed thoroughly. Thus, this study aims to highlight, through a case report, the importance of dentists or oral and maxillofacial surgeons in the diagnosis and treatment of maxillofacial infections.

## 2. Case History

**Figure 1:**
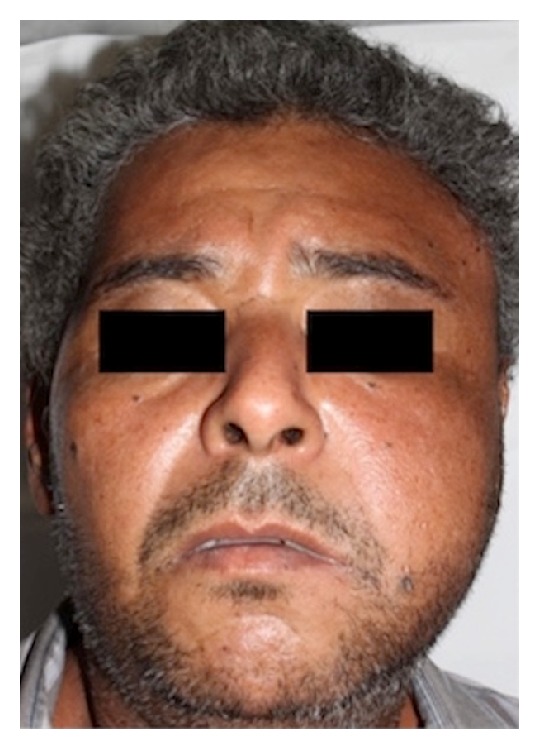


**Figure 2:**
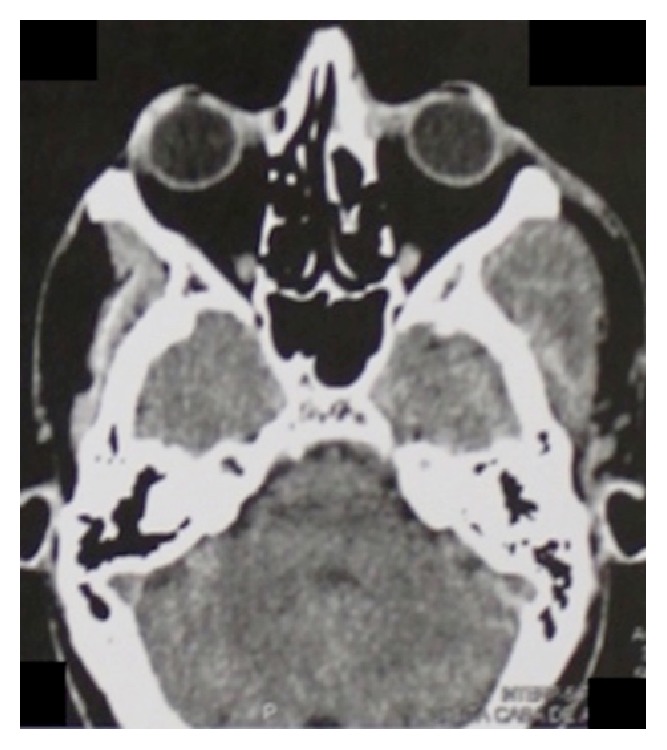


**Figure 3:**
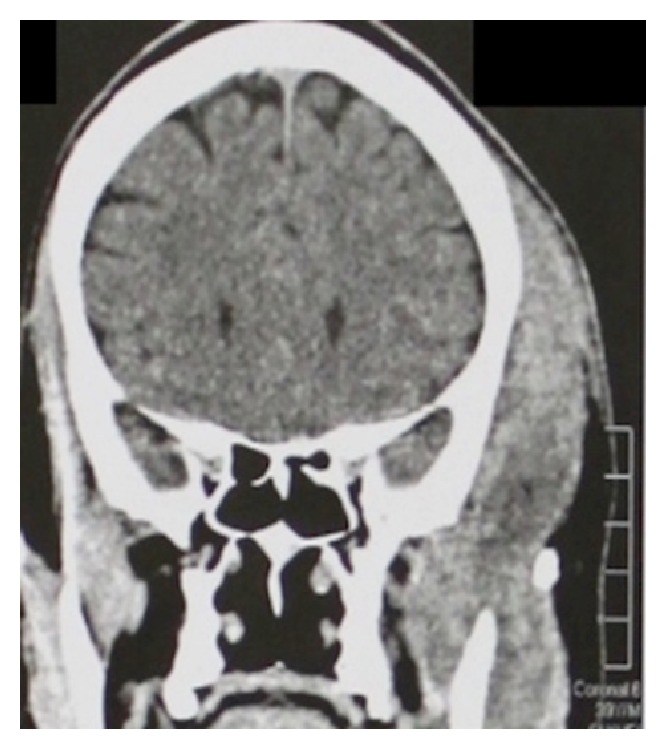


A 46-year-old male patient attended the emergency room of the* Santa Casa de Misericordia de Araçatuba* Hospital,* SP*, complaining about facial pain and swelling (Figure 1). On physical examination, left hemiface asymmetry and trismus were observed, while the facial CT examination showed a hyperdense mass involving the left masseteric, pterygomandibular, and superficial temporal regions (Figures 2 and 3). The patient was then referred to oral oncology center with cancer suspicion. However, he did not attend the oral oncology center.

**Figure 4:**
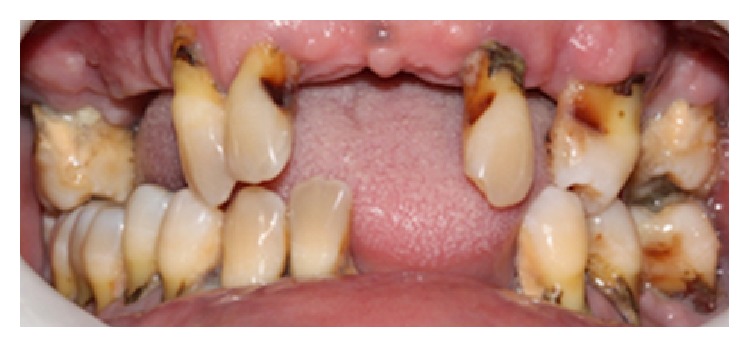


After 15 days, the patient returned to the same emergency room and was attended by the surgical and maxillofacial trauma team, presenting tachycardia, tachypnea, dysphagia, sore throat and breathing difficulty, trismus, and edema with floatation point in the temporal region. During anamnesis, the patient reported being uncontrolled diabetic, so a quick glucose test was performed noting a 600 mg/dL glucose level. In intraoral exam, a poor oral condition and generalized periodontitis were observed (Figure 4). The diagnosis was a complex odontogenic infection involving the masseteric, pterygomandibular, and superficial temporal spaces, confirmed by computed tomography.

**Figure 5:**
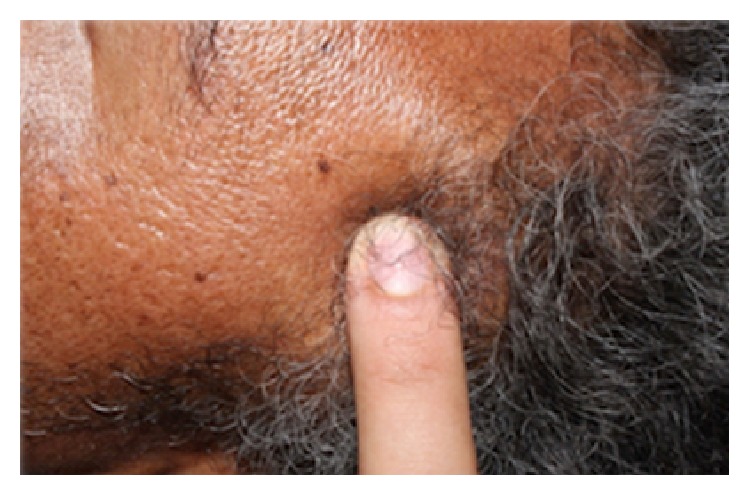


**Figure 6:**
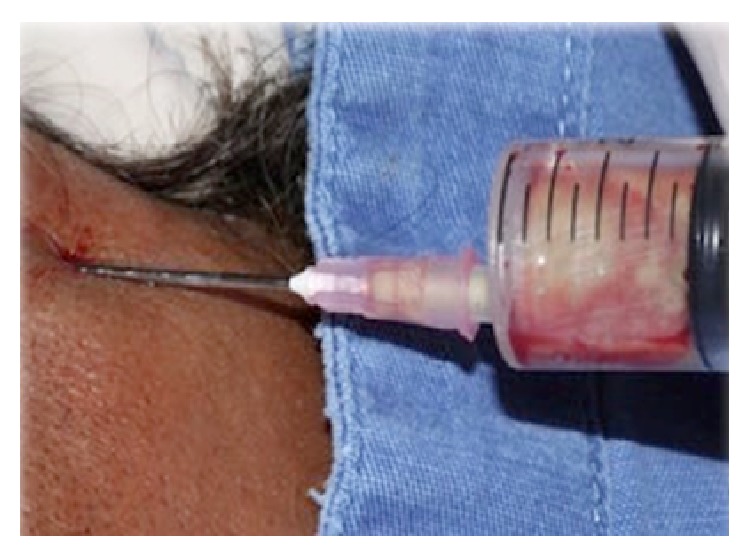


**Figure 7:**
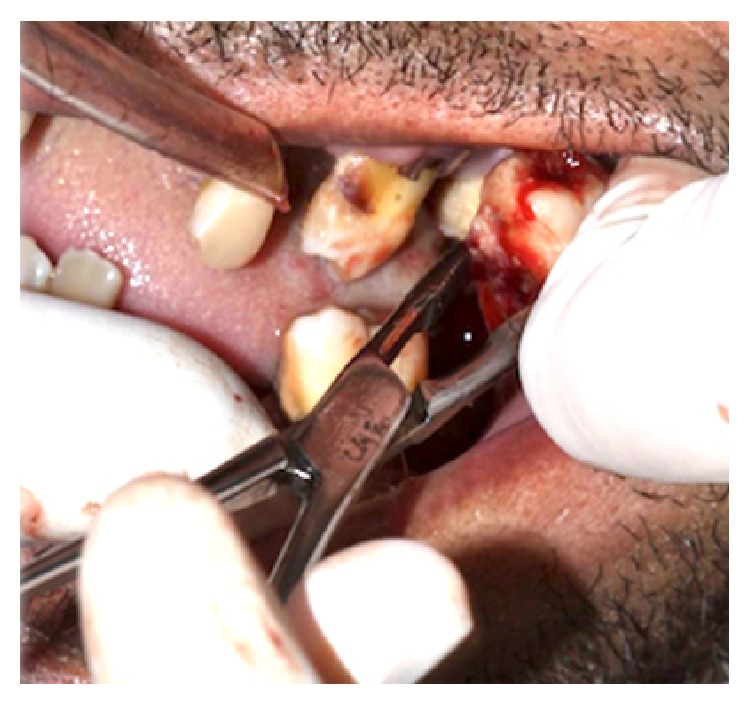


**Figure 8:**
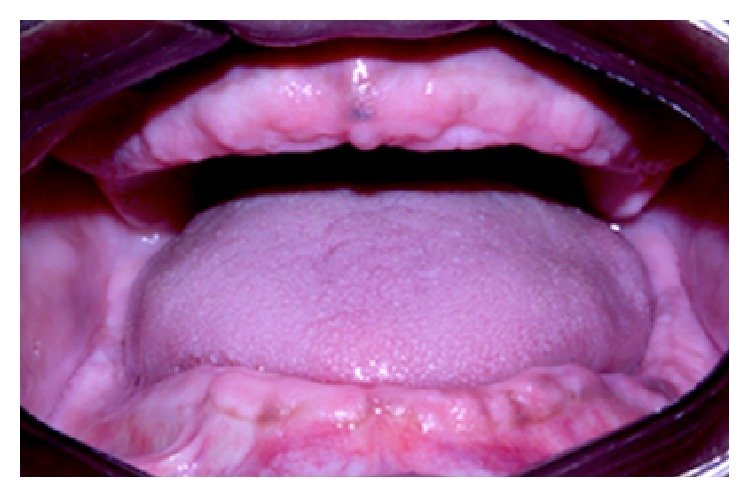


**Figure 9:**
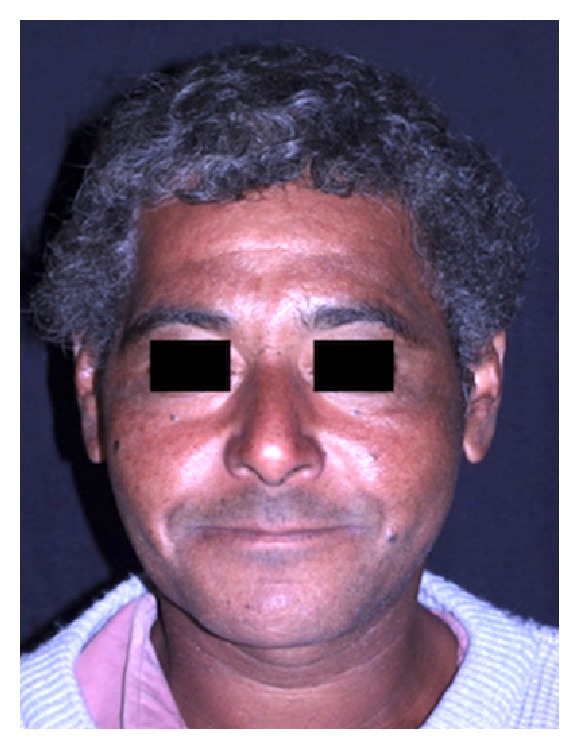


The treatment started immediately with intravenous antibiotic prophylaxis with 2 g sodium cephalothin (Keflin, União Química, Brasilia, DF, Brazil) and 500 mg metronidazole (Sanofi-Aventis Pharmaceuticals Ltd., Sao Paul, SP, Brazil) and administration of 10 IU insulin. The surgical procedure was conducted under general anesthesia and initiated by the location of floatation point in temporal region (Figure 5), where an aspiration punction for antibiogram culture and surgical drainage of superficial temporal space by Gillies technique with installation of a Penrose drain were performed (Figure 6). Two oral incisions were performed to access the masseteric and pterygomandibular spaces. Then, because of periodontal conditions and social-cultural aspect of the patient, a serial extraction was indicated (Figures 7 and 8). On the second day after surgery, the patient presented a good general condition and good mouth opening. The antibiotic therapy was 1 g ceftriaxone and was continued for 48 hours after symptom remission. The patient was referred to a dentist for rehabilitation with total prosthesis (Figure 9).

## 3. Discussion

Dental infection starts with pulp necrosis, which is disseminated to tissues through the apical foramen, reaching areas distant from the infection focus [8]. In a study with 185 patients suffering from deep neck infection, 50% of the cases were odontogenic [9], such as the case reported in our study. The current aspect of diagnosis and treatment of head and neck infections has improved due to technological advances in imaging and different types of antibiotics [10]. However, there is a low incidence of complications in patients with predisposing diseases for the development of infectious processes [5].

Uncontrolled diabetes mellitus can lead to high blood glucose levels, which contribute to a more aggressive infection course, owing to a deficiency in chemotaxis, phagocytosis, and bactericidal function. These factors predispose long periods of hospitalization increasing the risk of complications [7]. According to this report, a 600 mg/dL glycemic rate exacerbates infectious process and reduces pharmacological effect of antibiotics, making glycemic blood control a key factor in the cure of dental infection.

The clinical signs and symptoms of facial infection are remarkable, especially in superficial areas such as temporal and buccal space, clinically observed as a pronounced volumetric increase in the region [2]. However, some patients who exhibit milder signs may underestimate the case severity. Among the facial areas, when pterygomandibular space is affected, it should be treated aggressively, due to the possibility of contamination passing the edge of medial pterygoid muscle reaching the pharyngeal space [2].

Although not necessary in superficial infections, the CT scan is extremely valuable when the infection reaches deep spaces and when associated with an experienced clinical examination it is possible to identify clinically important pus cavities [1]. However, an evaluation performed by a nonspecialized professional can lead to misdiagnosis.

The cure of dental infections is usually achieved without major complications. Nevertheless, it is recommended that the dentist/doctor includes oral cavity as one of the main areas to be evaluated on physical examination in patients with painful edema in the head and neck region, investigating possible associated diseases that may be supporting the infection.

A problem in emergency attendance is the lack of knowledge from the side of the physician about dentistry comorbidities. Most misdiagnoses of head and neck infections happened due to first clinical examination failure; sometimes, it can be confused with tumor lesion. In this case, the first attendance led to wrong diagnosis and it could promote several complications, including death. Therefore, an oral and maxillofacial surgeon is essential to emergency room.

## References

[1] Gonzalez-Beicos A., Nunez D. (2012). Imaging of acute head and neck infections. *Radiologic Clinics of North America*.

[2] Flynn T. R. (2000). The swollen face: severe odontogenic infections. *Emergency Medicine Clinics of North America*.

[3] Wabik A., Hendrich B. K., Nienartowicz J., Guziński M., Sąsiadek M. J. (2014). Odontogenic inflammatory processes of head and neck in computed tomography examinations. *Polish Journal of Radiology*.

[4] Jundt J. S., Gutta R. (2012). Characteristics and cost impact of severe odontogenic infections. *Oral Surgery, Oral Medicine, Oral Pathology and Oral Radiology*.

[5] Opitz D., Camerer C., Camerer D.-M. (2015). Incidence and management of severe odontogenic infections—a retrospective analysis from 2004 to 2011. *Journal of Cranio-Maxillofacial Surgery*.

[6] Ylijoki S., Suuronen R., Jousimies-Somer H., Meurman J. H., Lindqvis C. (2001). Differences between patients with or without the need for intensive care due to severe odontogenic infections. *Journal of Oral and Maxillofacial Surgery*.

[7] Chang J. S., Yoo K. H., Yoon S. H. (2013). Odontogenic infection involving the secondary fascial space in diabetic and non-diabetic patients: a clinical comparative study. *Journal of the Korean Association of Oral and Maxillofacial Surgeons*.

[8] Saito C. T. M. H., Gulinelli J. L., Marão H. F. (2011). Occurrence of odontogenic infections in patients treated in a postgraduation program on maxillofacial surgery and traumatology. *The Journal of Craniofacial Surgery*.

[9] Huang T.-T., Liu T.-C., Chen P.-R., Tseng F.-Y., Yeh T.-H., Chen Y.-S. (2004). Deep neck infection: analysis of 185 cases. *Head & Neck*.

[10] Uluibau I. C., Jaunay T., Goss A. N. (2005). Severe odontogenic infections. *Australian Dental Journal*.

